# Pilot Demonstration of a Strengthening Method for Steel-Bolted Connections Using Pre-Formable Carbon Fiber Cloth with VaRTM

**DOI:** 10.3390/ma14092184

**Published:** 2021-04-24

**Authors:** Takahiro Matsui, Kohei Suzuki, Sota Sato, Yuki Kubokawa, Daiki Nakamoto, Shijir Davaakhishig, Yukihiro Matsumoto

**Affiliations:** 1 ACM Technology Department, Toray Industries, Inc., 2-1-1 Nihonbashimuromachi, Chuo-ku, Tokyo 103-8666, Japan; takahiro.matsui.f3@mail.toray; 2 Technical Development Headquarters, Constec Engi, Co., Ltd., 6-1-1 Heiwajima, Ota-ku, Tokyo 143-0006, Japan; suzuki-kohei@cons-hd.co.jp (K.S.); sato-sota@cons-hd.co.jp (S.S.); 3 Department of Architecture and Civil Engineering, Toyohashi University of Technology, 1-1 Hibarigaoka, Tempaku-cho, Toyohashi, Aichi 441-8580, Japan; kubokawa.yuki.vo@tut.jp (Y.K.); nakamoto.daiki.md@tut.jp (D.N.); shijir.davaakhishig.hj@tut.jp (S.D.)

**Keywords:** carbon fiber, preform, VaRTM, CFRP, strengthening, steel structure

## Abstract

In recent years, many seismic retrofitting methods have been performed to improve the structural performance and prevent the brittle failure of structural members. In the case of steel structures, slender seismic braces have been widely used for buildings, towers, and bridges. The brace connections should resist the full plastic axial tension load to ensure adequate plastic deformation performance for vibration energy absorption. However, certain connections do not satisfy these requirements. Recently, carbon fiber reinforced plastic (CFRP) has been used extensively to strengthen existing structures because of its high-strength, high elastic modulus, and light-weight characteristics. In this paper, we investigate the applicability of CFRP strengthening for brace connections and gusset plates with stepped surfaces using the vacuum-assisted resin transfer molding technique as a pilot demonstration. Stepped surfaces can be eliminated by using alternative CFRP layers to straighten the structural CFRP layers in order to effectively transfer the axial stress. Eventually, it is shown that CFRP strengthening can improve the connection strength and plastic deformation with 3% elongation, even if the CFRP is molded on the stepped surface.

## 1. Introduction

### 1.1. Research Background

Steel structures have excellent ductile performance due to their plastic deformability, in addition to having good seismic resiliency and energy absorption. Thus, steel materials have been widely used for building structures, latticed towers, large span bridges, high-rise buildings, and spatial structures. On the other hand, to prevent structural damage caused by severe earthquakes [1,2], seismic retrofitting has been rapidly applied to existing structures [3,4]. In the case of steel structures, it has been reported that several seismic brace members lack the connection strength—especially in terms of the load-carrying capacity of the net area at the bolt hole and the gusset plate connection—needed to reach axial full plastic capacity [5]. Although energy absorption via plastic deformation is considered in the seismic design of steel braces, brittle failure in the net area without plastic deformation of the gross sectional area often occurs. Therefore, several strengthening methods have been proposed to prevent brittle failure in the net area [6]. Member ductility of 8.5 is recommended to satisfactory perform seismic energy absorption.

To ensure the seismic performance of steel structures, several approaches can be taken. Sarno et al. [3] investigated the effects of concentrated bracing, mega-brace systems, and buckling restrained braces (BRBs) using nonlinear time history response analyses. Tena-Colunga and Vergara [7] suggested that BRBs have better seismic retrofitting effects compared with traditional bracing systems; however, the installation costs for BRBs are increased, as shown by comparing studies of the effects and costs of bracing systems and BRBs for seismic retrofitting. Usami et al. [4] showed the performance of BRBs experimentally, and their effective seismic retrofitting effects for arch bridge were represented analytically. Many studies have pointed out that bracing systems can provide higher seismic resistance through their effective horizontal resistance. However, sufficient connection strength is required to ensure the strength and ductility of the brace member. Cui et al. [5] experimentally and analytically reviewed the strength of gusset plate connections with several different geometry plates under axial tension load. They suggested that the design recommendation by the Architectural Institute of Japan (AIJ) [8] can be used to evaluate the connection strength within safe value ranges. Tremblay [9] experimentally investigated the seismic behavior of bracing systems with various cyclic loads, brace member shapes, and compressive strengths, whereby post-buckling resistance and design equations were proposed. Additionally, the Japan Building Disaster Prevention Association (JBDPA) established evaluation guidelines for seismic performance for the inspection and retrofitting of existing steel structures [6] to protect them from severe seismic damage [1]. Kasai et al. [10] developed repair methods for existing beam–column connections using bolted connections to prevent failure of welded connections at the edges of beams.

Almost all retrofitting methods for existing steel structures require bolted or welded connections in order to install the alternative members. However, the welding of steel plates requires high-temperature tools, and certain industrial plants, chemical plants, and factories cannot allow the use of high-temperature tools because of the risk of fire. The additional of bolt holes requires heavy tools, and there is often of a lack of space to set up such tools. Moreover, strengthening using steel increases the weight of structures and its related increasing seismic loads.

On the other hand, strengthening and repair methods using carbon fiber reinforced plastic (CFRP) have been gradually applied to existing steel structures [11,12,13] with insufficient structural performance in aging or deteriorating structures. CFRP has good mechanical and physical characteristics, e.g., high-strength, high elastic modulus, lightweight, and good corrosion resistance performance. Moreover, CFRP can be conveniently connected to steel using adhesive bonding, which can reduce the need for heavy, high-temperature tools. Thus, CFRP can be easily used in construction without requiring any heavy machines and can minimize the weight of the resulting strengthened member. Almost all on-site CFRP strengthening methods use adhesively bonded connections on steel surfaces in order to apply CFRP, either by adhesively bonding CFRP plates [11,12,13,14,15] or hand-layup molding and bonding of carbon fiber cloth [11,16,17,18,19,20]. However, CFRP plates can only be applied to planar surfaces because CFRP cannot be deformed so as to adjust it to curved and bumpy surfaces. Additionally, the on-site hand-layup molding method can be applied to various surface conditions; however, it requires lengthy construction time because the resin must be injected into carbon fiber cloth layer-by-layer, with the time increasing exponentially as the number of carbon fiber layer increases.

Based on the above, the authors developed an alternative CFRP strengthening method using an on-site vacuum-assisted resin transfer molding (VaRTM) technique with adhesive bonding for steel structures [21]. This technique the authors proposed can adhere and mold at the same time to strengthen existing steel structures. VaRTM methods have been used for the manufacture method of FRP products, without bonding to other materials, in blades of wind power generation facilities, in aerospace structures, and civil structures because of their excellent mechanical properties and short molding times. In this paper, we focus on steel brace connections for CFRP strengthening to meet seismic retrofitting requirements by VaRTM bonding. First, we clarify the strength requirements for steel brace connections. Next, a CFRP strengthening design method using VaRTM is proposed. Then, we demonstrate the proposed CFRP strengthening method and investigate the strengthening effects. Finally, it is shown that the proposed CFRP strengthening method can be successfully applied to steel brace connections, even for stepped and bumpy bonding surface, showing that the load-carrying capacity of the connections and ductility of the brace members can be effectively improved.

### 1.2. Strengthening Method Using CFRP by VaRTM

**Figure 1:**
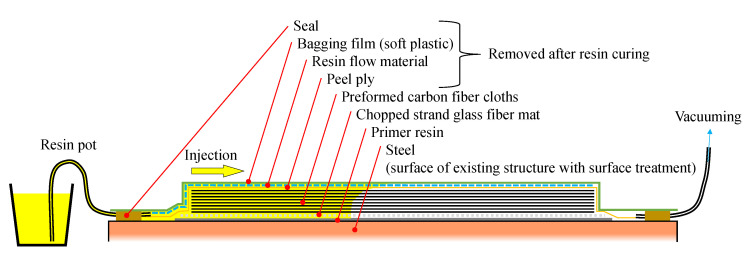
CFRP strengthening method by VaRTM.

**Figure 2:**
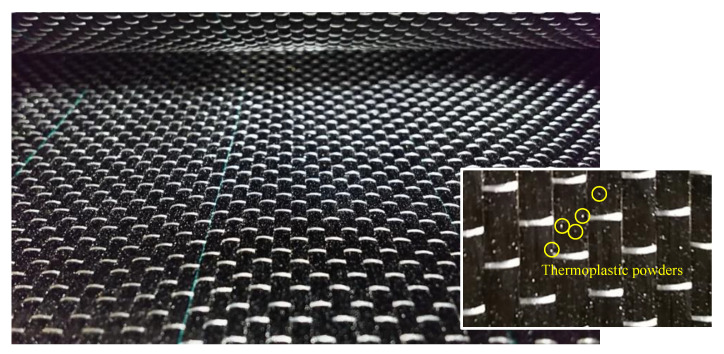
Pre-formable carbon fiber cloth.

VaRTM has been applied to mold large FRP products, such as the blades of wind turbines and monocoque automobiles and footbridges [22], because of its productivity, quality, and usability. The authors have developed an adhesively bonding and strengthening method by VaRTM through the modification of the VaRTM technique. Figure 1 shows a simplified diagram of the adopted CFRP strengthening method using VaRTM that the authors developed [21]. Usually, metallic frames are used to mold FRP products by VaRTM, and the frames are dismounted after curing. On the other hand, the adopted CFRP strengthening method using VaRTM uses existing steel surface as a molding frame. First, the steel surface is treated to remove paint and make a rough surface for adhesive bonding. It has been recommended that the surface roughness should be higher than 20 micro-strain of ten-point mean roughness [21]. Second, primer resin is applied to the steel surface to avoid galvanic corrosion by carbon fiber and steel. After curing of the primer resin, carbon fiber cloth ais set up onto the steel surface. Next, the molding utilities for VaRTM (peel ply, resin flow material, seal, and bagging film) are attached. Finally, vacuuming and resin injection are carried out. By using this method, carbon fiber cloth can be molded and bonded directly onto various steel surfaces, even if many carbon fiber cloths are required for strengthening. Thus, the molding time can be greatly shortened, and stable mechanical performance can be obtained compared to the hand-layup molding method. Additionally, carbon fiber cloth can be easily molded for vertical surfaces, such as steel brace connections. Moreover, pre-formable carbon fiber cloth has been developed in recent years to allow molding with a large number of carbon fiber cloth layers and to reduce the imperfections caused by cloth slippage. To make the pre-formable carbon fiber cloth, thermoplastic powder was applied onto carbon fiber cloth, as shown in Figure 2. Thus, the cloth can be tentatively formed by heating because thermoplastic can be remolded easily by heating. In this study, we stacked carbon fiber cloth in layers and unified them by heating to ease the setup of the carbon fiber cloth on the steel surface. The details of the strengthening procedure for the targeted structure in this research are introduced in Section 2.3.

## 2. Experimental Methods

This section shows the design requirements for seismic resisting steel braces, not only in terms of load-carrying capacity, but also in terms of ductile performance. The calculation method was used to predict of the number of layers of carbon fiber cloth needed to avoid a stepped surface and to transfer axial force.

### 2.1. Seismic Resisting Steel Braces and Its Seismic Performance Evaluation

The Japan Building Disaster Prevention Association (JBDPA) established an evaluation guideline for the seismic performance of existing steel structures [6]. According to their method, the load-carrying capacity $P_{u}$ of a seismic brace for existing steel structures under tensile force can be evaluated using the following equations:

(1) $$P_{u}=\min\left\{P_{1},\;P_{2},\;P_{3},\;P_{4}\right\}$$

- (1) Ultimate shear strength of bolts:
  (2) $$P_{1}=0.6\times m\times n\times A_{b}\times_{f}F_{u}$$

where, 

m is a total number of shear planes;

n is a total number of bolts;

$A_{b}$ is sectional area of bolt;

${}_{f}F_{u}$ is tensile strength of bolt (1000 N/mm^2^).

**Figure 3:**
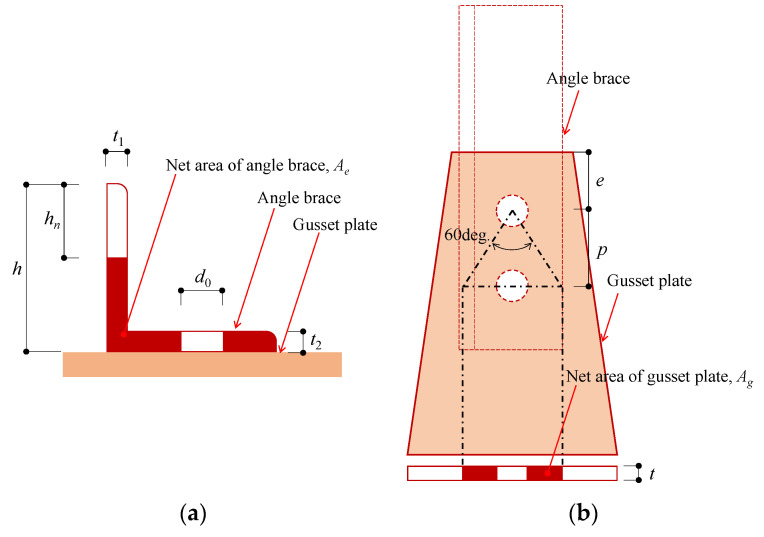
Variables used to calculate tensile strength of brace in net area: (**a**) net area of brace; (**b**) net area of gusset plate.

- (2) Tensile strength of brace in net area:
  (3) $$P_{2}=A_{e}\times F_{u}=\left(A-d_{0}t_{2}-h_{n}t_{1}\right)n_{B}\times F_{u}$$

where, 

$A_{e}$ is a net area of brace;

$F_{u}$ is tensile strength of brace (400 N/mm^2^ for SS400 steel);

A is sectional area of brace; 

$d_{0}$, $t_{2}$, $h_{n}$, and $t_{1}$ are shown in Figure 3 ($h_{n}$ = 0.7 for n = 2);

$n_{B}$ is a total number of braces.

- (3) Shear-out strength of brace or gusset plate:
  (4) $$P_{3}=\left\{e+\left(m-1\right)p\right\}n\times t\times F_{u}$$

where, 

e is end distance;

m is a number of bolts on axial direction;

p is bolt pitch;

n is a number of bolts on transverse direction;

t is thickness of brace or gusset plate;

$F_{u}$ is tensile strength of brace or gusset plate (400 N/mm^2^ for SS400 steel).

- (4) Ultimate strength of gusset plate in net area:
  (5) $$P_{4}=A_{g}\times F_{u}$$

where,

$A_{g}$ is net area of gusset plate.

On the other hand, the JBDPA requires that the connection strength satisfies the following strength to resist the full plastic axial load-carrying capacity and large plastic deformation with hysteresis energy absorption:

(6) $$P_{u}\geq P_{req}=1.2\times A\times F$$

where, 

A is sectional area of brace;

F is nominal yield stress of steel (235 N/mm^2^ for SS400 steel).

**Figure 4:**
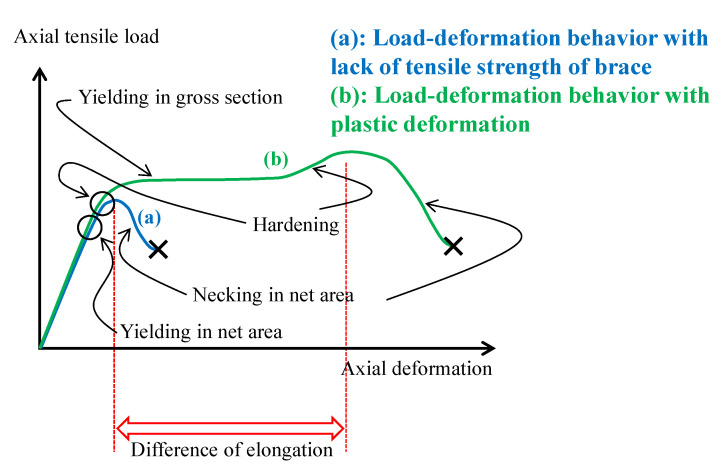
Load–deformation relations with or without plastic deformation.

**Figure 5:**
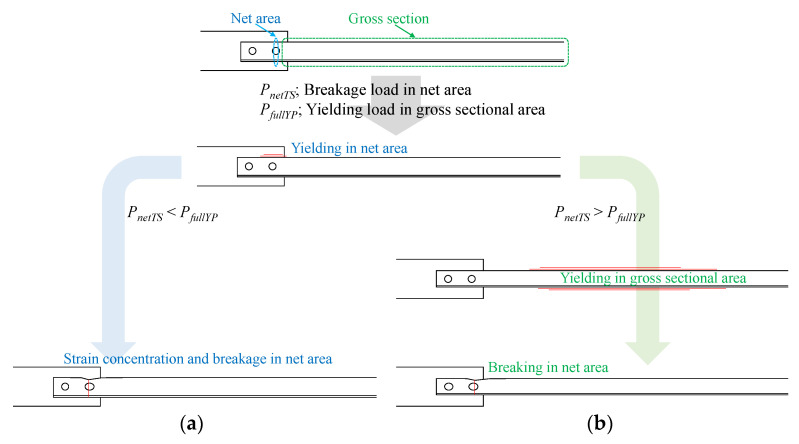
Plastic behavior of steel brace: (**a**) lack tensile strength brace; (**b**) enough tensile strength brace.

However, several existing steel brace connections designed using older Japanese seismic design codes do not satisfy the JBDPA requirements, especially in terms of the tensile strength of the brace in net area $P_{2}$. This is because satisfaction of Equations (1)–(6) was not required for structural design of the brace connections. In the case of a lack of tensile strength of the brace in the net area, breakage occurs without ductile behavior, as shown in Figure 4 and Figure 5a. Therefore, extensive brittle failure has been reported in severe earthquakes [23]. On the other hand, although sectional loss from bolt holes occurs at the connections, yielding in gross sectional areas can be observed when the breakage load in the net area is higher than the yielding load at the gross sectional area, as shown in Figure 4 and Figure 5b. Thus, several retrofit methods have been proposed, and seismic retrofitting for net areas has been rapidly applied according to the evaluation and retrofitting guidelines [6]. However, previous retrofitting method have required welding or alternative bolt holes being added on site to connect additional steel elements. This means that heavy materials and tools must be used, especially as welding cannot be conducted in certain factory buildings due to fire risks.

### 2.2. Specimens

**Figure 6:**
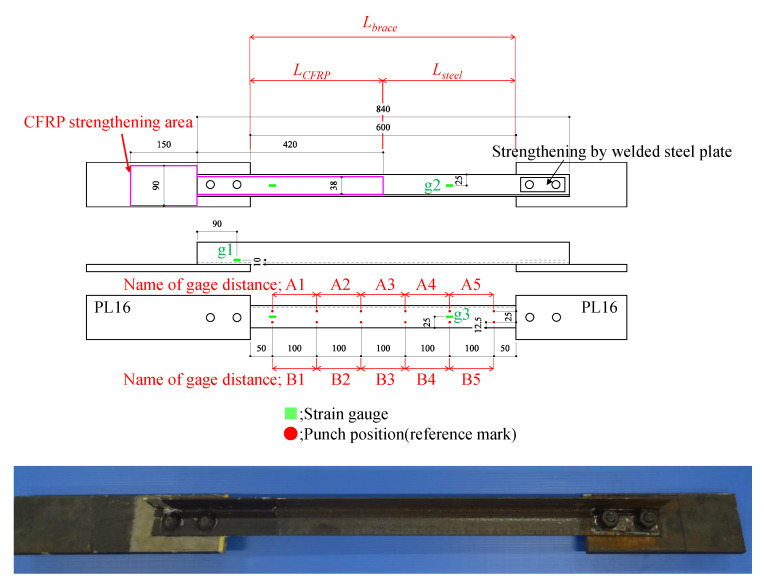
Geometry of specimen.

Based on the above, we adopted a steel-bolted connection with a lack of tensile strength in the net area, as shown in Figure 6. We adopted SS400 angle steel with a 50 × 50 × 4 mm^3^ sectional size as a brace and SS400 steel plates of 16 mm thickness and 100 mm width as gusset plates. Several punch marks of 100 mm pitch were created on the steel brace to evaluate plastic deformation after testing, as shown in Figure 6. M16 high-strength steel bolts were used to connect the brace and gusset plates. The minimum yield stress and tensile strength values for SS400 steel is 245 N/mm^2^ and 400 N/mm^2^, respectively [24]. The steel plate around the bolt holes was welded to prevent failure in the net area at the right-side connection. Table 1 shows the load-carrying capacity as evaluated using Equations (2)–(6). Thus, the specimen will break in the net area without plastic deformation in the gross section.

Table 2 shows the experimental parameters—without CFRP strengthening specimen (namely NS) and two type of CFRP strengthening specimens (namely CFS-full and CFS-loss). We adopted two types of CFRP strengthening—the gross sectional strengthening model and sectional loss strengthening model. The total number of CFRP layers for the gross sectional strengthening model was calculated by the strength of the gross sectional area of steel brace, while the sectional loss model multiplied the sectional area of the bolt hole by the strength of the steel. Thus, CFRP of the gross sectional strengthening model has an equivalent strength of steel brace, while the CFRP strength of the sectional loss strengthening model corresponds to the strength of lost area of steel by bolt hole. Equations (7) and (8) show the calculation method of total number of CFRP layers for stress transfer layer.

(7) $$\mathrm{Gross}\;\sec\mathrm{tional}\;\mathrm{strengthening}\;\mathrm{model};\;n_{ST}\geq \frac{A\times F_{u}}{t_{UM}\times b_{CFRP}\times F_{UM}}$$

(8) $$\mathrm{Sec}\mathrm{tional}\;\mathrm{loss}\;\mathrm{strengthening}\;\mathrm{model};\;n_{ST}\geq \frac{t_{2}\times F_{u}}{t_{UM}\times F_{UM}}$$

$F_{UM}$: tensile strength of carbon fiber (2400 N/mm^2^)

We adopted a unidirectional, medium-elasticity carbon fiber cloth designated as UM46-40P (Toray Industries, Tokyo, Japan) and a bi-directional, high-strength carbon fiber cloth, BT70-20 (Toray Industries, Tokyo, Japan), to strengthen steel connections. UM46-40P carbon cloth was used for removing steps and bumps, and for stress transfer. The BT70-20 bi-directional carbon cloth was used for improvement of the bonding area on the gusset plate because the gusset plate is usually wider than the brace. UM46-40P carbon fiber cloth is a commercial product, which has an adhered thermoplastic powder on the surface to enable preliminary forming by heating, as shown in Figure 2.

Table 3. shows the specifications for the steel and carbon fiber cloths.

**Figure 7:**
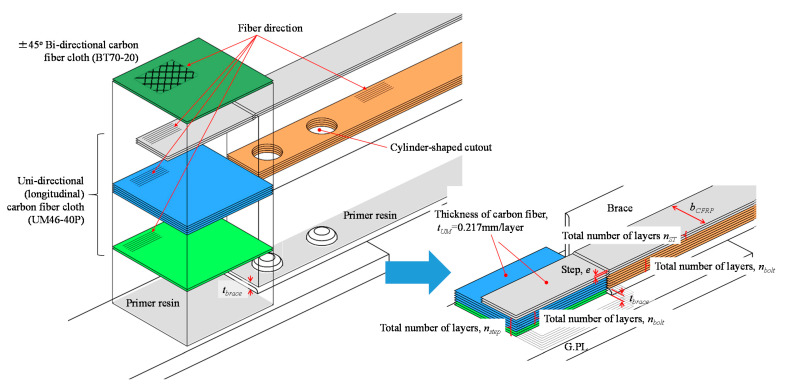
Strengthening method diagram and variables in Equations (7) and (8).

In the steel bolted connection, stepped surfaces are commonly found, as shown in Figure 7. To strengthen a steel connection with a stepped surface, the step between the gusset plate and brace surface should be minimized to reduce the bending force applied to the CFRP layer. Additionally, the CFRP should transfer the axial force to prevent strain concentration around the bolt hole. Thus, we decided on the number of carbon fiber layers needed to minimize step e using Equation (9). Next, the axial and bending stress values for CFRP under axial force *N* were evaluated by Equation (10). The variables in Equations (9) and (10) are also shown in Figure 7. To determine the number of carbon fiber layers with fiber volume content $V_{f}$ variation, we assumed that the fiber volume content $V_{f}$ would vary by 45% to 55% based on the previous research [21]. BT70-20 bi-directional carbon cloth is used on outermost layers to expand the width of the bonding area and effectively transfer the stress widely because the gusset plate is usually wider than the brace.

(9) $$e=\left|t_{brace}-\frac{t_{UM}\left(n_{step}-n_{bolt}\right)}{V_{f}}\right|$$

(10) $$\sigma_{CFRP}=\frac{N}{b_{CFRP}t_{CFRP}}+\frac{Ne}{b_{CFRP}t_{CFRP}{}^{2}/6}=\frac{N}{b_{CFRP}n_{ST}t_{UM}/V_{f}}+\frac{Ne}{b_{CFRP}{\left(n_{ST}t_{UM}/V_{f}\right)}^{2}/6}$$

where,

*e* is remaining step after CFRP strengthening;

*t_brace_* is thickness of steel brace;

*t_UM_* is thickness of carbon fiber cloth UM46-40P;

*n_step_* is a total number of carbon fiber layers required for step minimization;

*n_bolt_* is a total number of carbon fiber layers required for bump minimization;

*V_f_* is carbon fiber volume content;

*b_CFRP_* is width of CFRP;

*t_CFRP_* is thickness of CFRP;

*n_st_* is a total number of carbon fiber layers for required axial force transfer.

**Figure 8:**
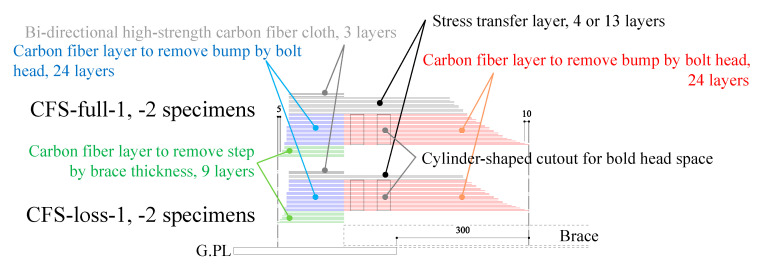
Lamination condition of CFRP strengthening.

Eventually, the total number of carbon fiber layers needed to minimize step $n_{step}$ was determined as 33 layers (9 layers to adjust the brace thickness, and 24 layers to adjust the height of the bolt head). The total number of carbon fiber layers needed to transfer the axial force $n_{ST}$ was determined as 13 layers for the gross sectional strengthening model and 4 layers for the sectional loss strengthening model. Figure 8 shows the lamination conditions. Bonding length is important to perform the strengthening effect and to avoid debonding failure. However, only a few hundred-millimeter bonding length can be used for the bonding on gusset plate. Thus, we decided the bonding length should be 150 mm for the gusset plate area because the actual brace connections lack the applicable surface area to adhesively bond the CFRP. However, gusset plates are wider than the brace width, so we applied wider carbon fiber cloth for the step adjustment and alternative bi-directional high-strength carbon fiber cloth on the outermost layer to expand the width of the bonding area shown in Figure 7. On the other hand, the brace members have sufficient length to allow for bonding, while it is known that the bonding capacity will peak with increasing the bonding length [18]. Thus, we decided the bonding length should be 300 mm for the brace side with a 150 mm uniform carbon fiber layer length and a 150 mm taper length based on the previous fundamental bonding test results [21].

### 2.3. Specimen Preparation

**Figure 9:**
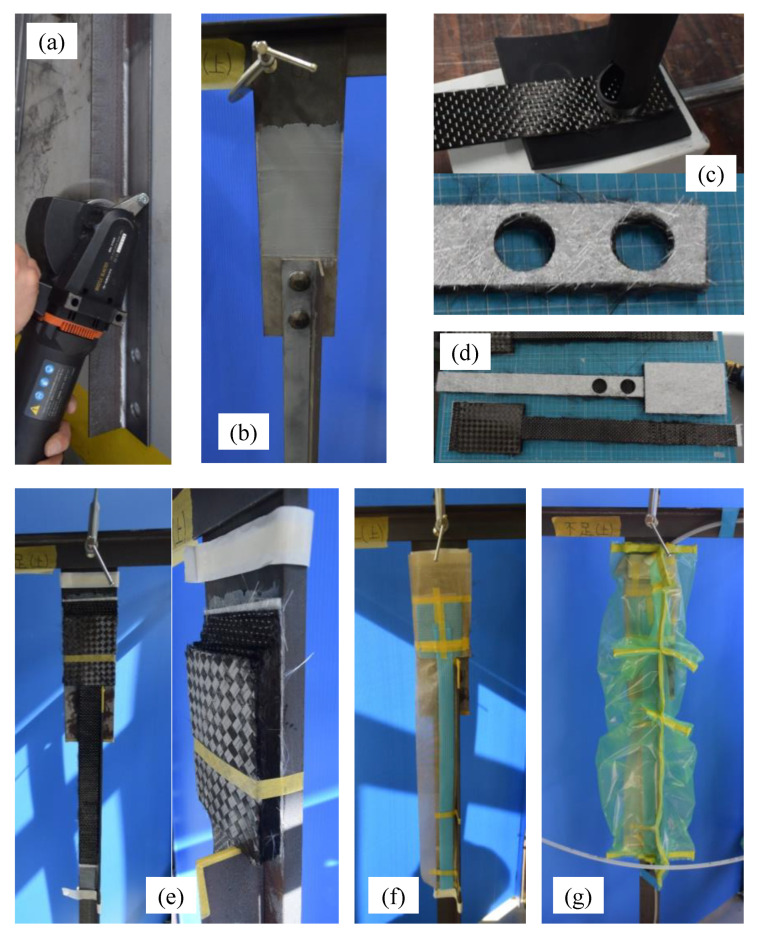
Molding and bonding process: (**a**) surface treatment; (**b**) application of primer resin; (**c**) cutout of carbon fiber cloth; (**d**) pre-formed carbon fiber cloths; (**e**) setup of preformed carbon fiber cloth; (**f**) setup of peel ply and resin flow media; (**g**) setup of bagging film around the specimen for vacuuming and resin injection.

Figure 9 shows the molding process. First, the steel surface was treated to make a rough surface using a blast surface forming power tool (Bristle Blaster^®^, G-TOOL Co., Aichi, Japan), as shown in Figure 9a. We set the minimum surface roughness as 20 micro-strain with ten-point mean roughness using a surface roughness tester SJ-210 (Mitsutoyo, Tokyo, Japan). Second, E258R room temperature curable primer epoxy resin (Konishi, Tokyo, Japan) was applied on the steel surface to improve the bonding strength between the steel and CFRP, as shown in Figure 9b. After curing the primer resin, preformed carbon fiber cloth (shown in Figure 9c) and molding materials were attached, and the molding area was covered by vacuum film, as shown in Figure 9d,e. Finally, AUP40T1 low-viscosity, room temperature curable epoxy resin for VaRTM bonding and molding (Toray Building Materials, Tokyo, Japan) was injected after the vacuum pressure and absence of leakage were confirmed. The conditions in Figure 4 correspond to the situation shown in Figure 9e.

**Figure 10:**
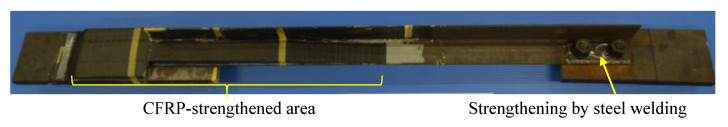
Specimen with CFRP strengthening.

Figure 10 shows the specimen with CFRP strengthening applied. The average fiber volume content was 50.4% with a 4.97% coefficient of variation, as measured by a SWT-9000 vortex current coating thickness meter with an Fe-20 probe (Sanko Electric Laboratory Co., Tokyo, Japan) after demolding.

## 3. Experimental Results

**Figure 11:**
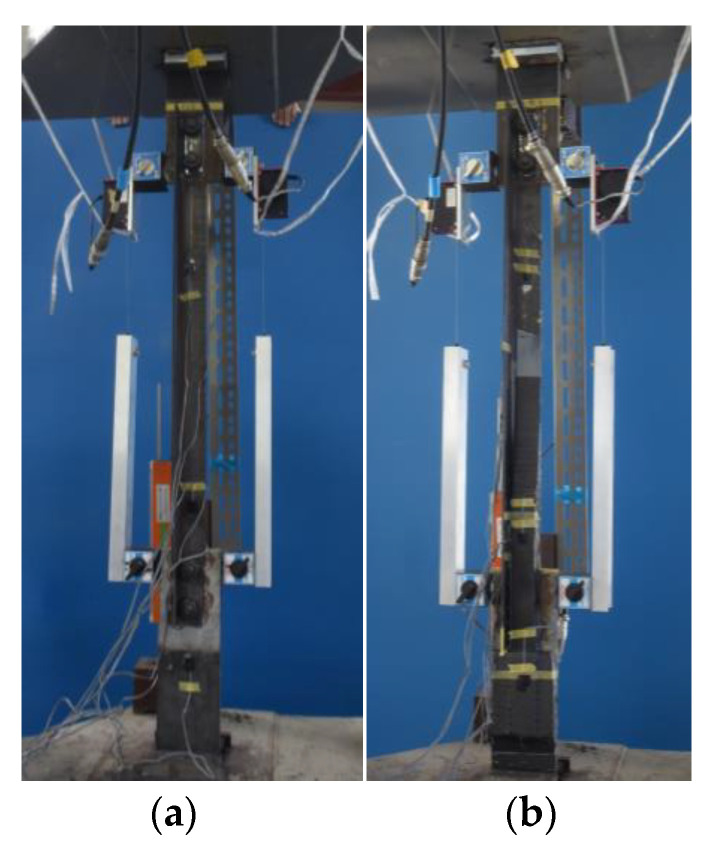
Experimental setup: (**a**) NS specimen; (**b**) CFS-loss specimen.

**Figure 12:**
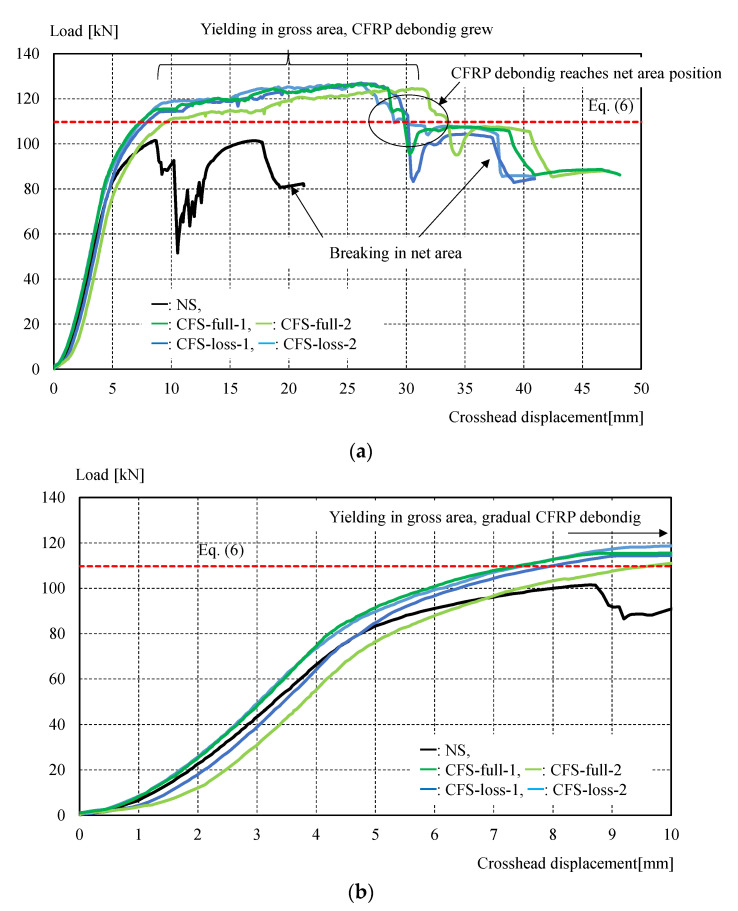
Load–crosshead displacement relations: (**a**) a range of 0–50 mm with experimental observation; (**b**) a range of 0–10 mm.

**Figure 13:**
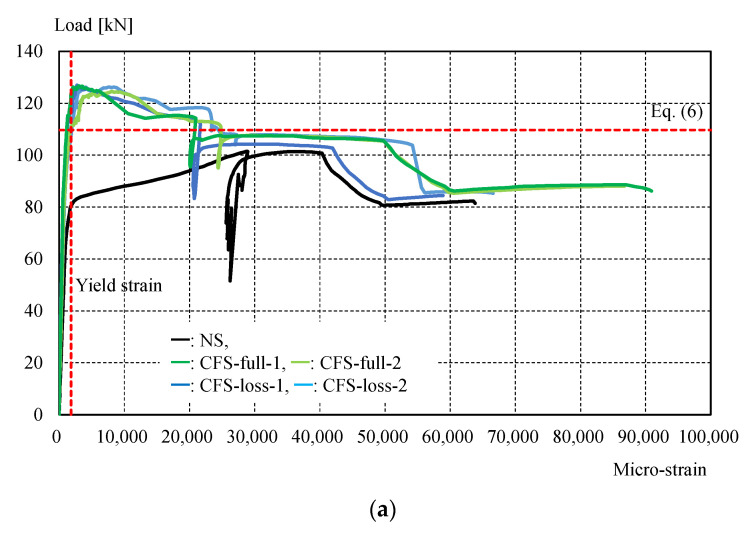

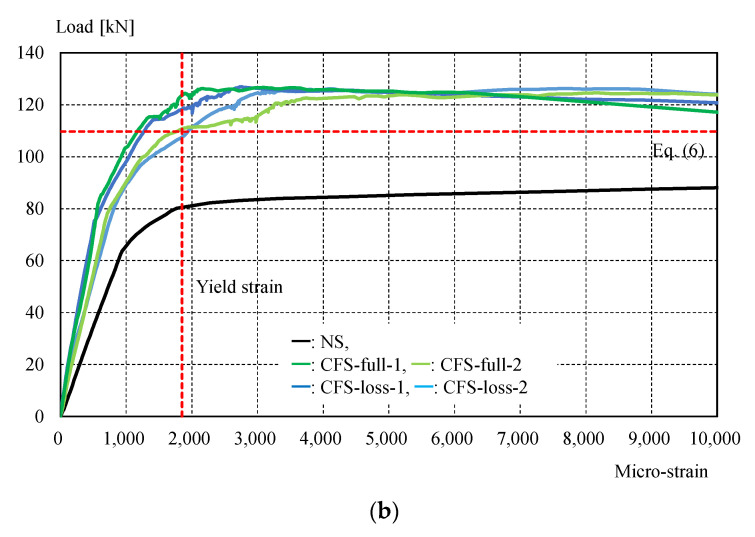
Load–strain (g1) relations: (**a**) a range of 0–10% strain; (**b**) a range of 0–1% strain.

**Figure 14:**
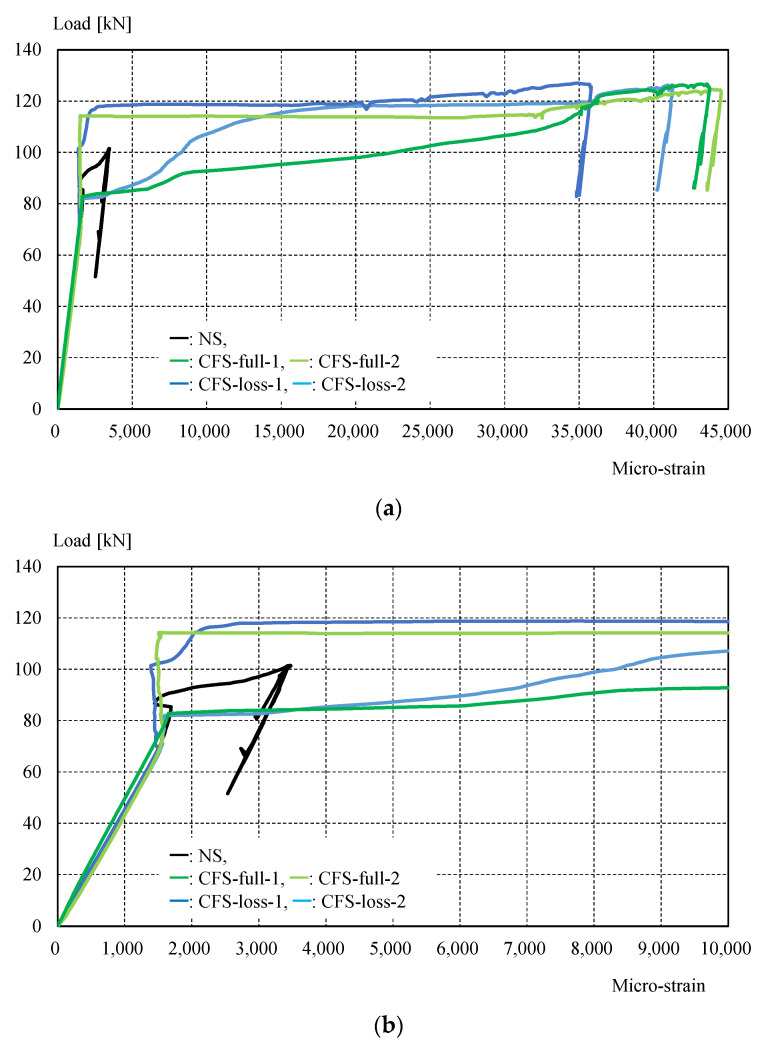
Load–strain (average of g2 and g3) relations: (**a**) a range of 0–4.5% strain; (**b**) a range of 0–1% strain.

**Figure 15:**
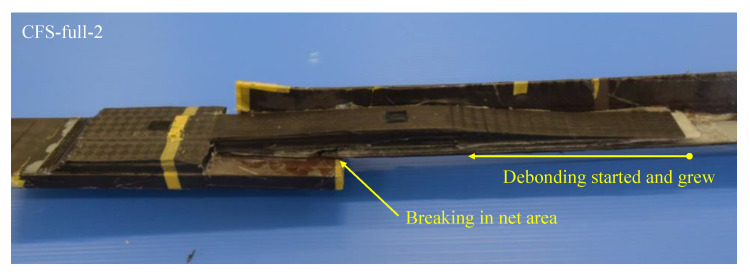
Typical failure mode of CFRP strengthening specimen.

Figure 11 shows the experimental setup, while Table 4 shows the maximum load and failure modes. All specimens were monotonically tested in a 1000 kN Shimadzu universal testing machine, as shown in Figure 11. A load cell built in the testing machine was used to measure the applied load. Displacement transducers were mounted to measure crosshead displacement, and the longitudinal strain in specimens was measured by strain gauges attached directly to the steel and CFRP in several positions shown in Figure 6. It was demonstrated that CFRP strengthening could effectively increase the load-carrying capacity. Figure 12 shows the load-crosshead displacement relations, with the evaluated strength calculated using Equation (6). CFRP strengthening could increase not only the load-carrying capacity, but also the elongation, because the displacement just before breakage in the net area could be significantly increased (shown in Figure 12) in all CFRP strengthening specimens. This means that ductile deformation behavior occurred because CFRP strengthening could cause yielding of the gross section earlier than the net area. On the other hand, a lack of plastic deformation and brittle failure occurred without energy absorption due to hysteresis if the full plastic axial load after hardening in the net area was lower than the yielding load in the gross section, as seen in specimen NS. Thus, the performance of CFRP strengthening reached previously established steel-welded strengthening methods [6] without fire risk and use of heavy tools. Furthermore, no significant changes in elastic stiffness could be found, as shown in Figure 12b. Figure 13 shows load–strain relations at position g1, which is close to the bolt hole. In the case of specimen NS, yielding was observed around 80 kN due to stress concentration, while plastic strain grew rapidly. However, in the case of the CFRP-strengthened specimen, elastic behavior could be maintained until 110 kN, without causing large plastic strain. Thus, it can be confirmed that the CFRP strengthening can effectively decrease the strain nearby the bolt hole and delay the yielding in the net area to perform yielding load in the gross area. After reaching the maximum load, the plastic strain grew due to CFRP debonding, and breakage in the net area finally occurred. Additionally, the loading required for ductility shown in Equation (6) was satisfied without yielding in the net area. Loadings in the breakage section of the net area stayed almost constant for all specimens, because the CFRP was debonded by the plastic strain. This is because the primer resin cannot resist large strains. Thus, there was no difference in mechanical conditions just before breakage in the net area between the NS and CFRP-strengthened specimens. Figure 14 shows load–average strain relations at positions g2 and g3. Slight plastic strain occurred in specimen NS because the loading was decreased by the stress concentration in the net area and the associated necking. On the other hand, values of more than 35,000 micro-strain were observed in CFRP strengthening specimens. This means that the gross section position of the steel brace can be elongated due to prevention of the stress concentration in the net area using CFRP strengthening until debonding, as also shown in Figure 13b. Figure 14b shows the yielding behavior of the brace in the gross area. In the case of the specimens CFS-full-1 and CFS-loss-2, yielding was observed at 80kN loading, while specimens CFS-full-2 and CFS-loss-1 yielded at 115 and 100 kN, respectively. This variation of behavior was affected by the strain gauge position, because Figure 13 shows a strain of quite a local position. Eventually, CFRP strengthening specimens resisted almost the same load carrying capacity of 125 kN, as shown in Table 4. Additionally, it was estimated that the CFRP strengthening effect vanished due to plastic strain measuring 35,000–450,000 micro-strain by the CFRP debonding. Thus, it is thought that elongation of the CFRP strengthening steel brace can account for 3% plastic deformation without debonding. Figure 15 shows the typical failure mode of the CFRP strengthening specimen. And Figure S1 (supplementary material) is an experimental video of CFRP strengthening specimen. It was observed that debonding began from the edge of the CFRP on the brace, and the debonding area was gradually grown.

**Figure 16:**
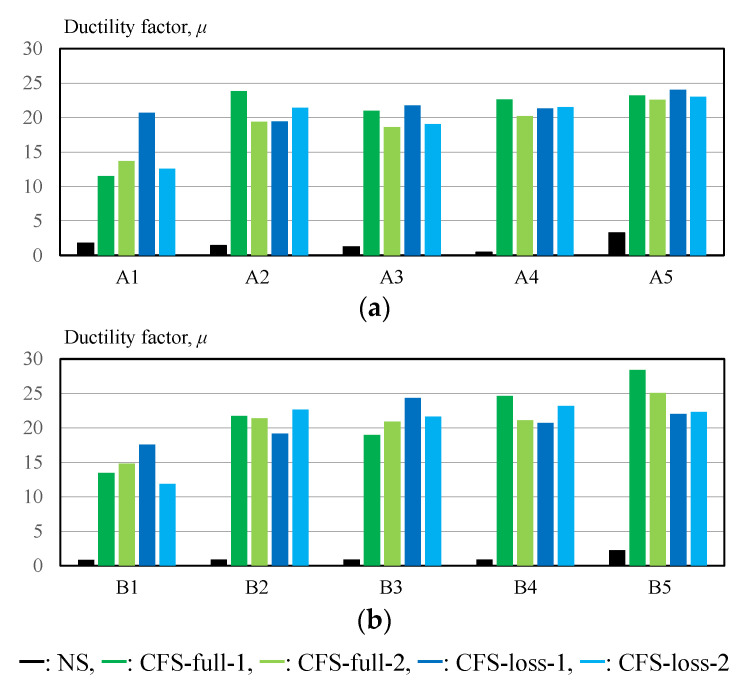
Ductility factor as evaluated through experiment: (**a**) gauge position A1 ~ A5; (**b**) gauge position B1 ~ B5.

Figure 16 shows the ductility factor, as calculated by the following equation:

(11) $$\mu =\frac{L_{1}-L_{0}}{L_{0}}/\frac{\sigma_{YP}}{E}+1$$

where, 

$L_{1}$ is gage distance after experiment;

$L_{0}$ is gage distance before experiment;

$\sigma_{YP}$ is yield stress of the steel brace (370 N/mm^2^);

E is elastic modulus of the steel brace (200 kN/mm^2^).

In the case of the NS specimen, the maximum value of the ductility factor was only 3.3 at position A5, as shown in Figure 6. On the other hand, the maximum ductility factor was 28 in specimen CFS-full-1, and the ductility factor values for all positions were larger than 11. In the case of CFRP-strengthened specimens, the ductility factor values decreased as the position approached the bolts. The ductility factor at the positions without CFRP strengthening (A4, A5, B4, and B5) reached 20–28. Thus, the total elongation can be calculated (including safety considerations) using the steel length $L_{steel}$ evaluated by subtracting the CFRP strengthening length $L_{CFRP}$ from the brace length $L_{brace}$, showing a maximum of 3% plastic deformation.

## 4. Conclusions

This paper proposed a CFRP strengthening method for steel brace connections using the VaRTM technique and demonstrated the effects of the proposed method. The VaRTM technique was improved as an adhesion and molding method to strengthen existing steel structures on site. The following findings were obtained:

1. We proposed a strengthening method using carbon fiber cloth for steel brace connections with steps and demonstrated that molding and bonding can be successfully conducted using VaRTM.
2. The load-carrying capacity of the connection and the ductility factor of the steel brace were significantly improved using CFRP strengthening.
3. The total elongation of the steel brace can be estimated using 3% plastic deformation of the length of bare steel position.

This method cannot cover all type of steel brace connections, because the various geometrical condition can be found on the connection. Thus, we will evaluate the applicability of these strengthening methods for the actual structures by finite element analysis and experiment, and the applicability, strengthening effects, strengthening design method, as well as experimental variation and stability will be clarified for the typical connection geometries in the future.

## Figures and Tables

**Table 1:** Evaluated load-carrying capacity values.

Failure Mode	Load-Carrying Capacity	Equation
Ultimate shear strength of bolts	241 kN	(2)
Tensile strength of brace in net area	70.8 kN	(3)
Shear-out strength of gusset plate	144 kN	(4)
Ultimate strength of gusset plate net area	576 kN	(5)
JBDPA requirement	110 kN	(6)

**Table 2:** Experimental parameters.

Specimen Name	Strengthening	Number of Specimens
NS	N/A	1
CFS-full-1, -2	Gross sectional strengthening model	2
CFS-loss-1, -2	Sectional loss strengthening model	2

**Table 3:** Material properties.

Material	Elastic Modulus	Yield Point	Tensile Strength
Steel (angle steel)	200 GPa *^1^	370 Mpa *^2^	457 Mpa *^2^
UM46-40P [25]	440 Gpa	-	2400 Mpa
BT70-20 [25]	230 GPa	-	2900 MPa

*^1^ Assumption (general value), *^2^ From manufacturer certificate.

**Table 4:** Maximum load and failure mode.

Specimen Name	Maximum Load	Failure Mode
NS	102 kN	Yielding and breaking at net area.
CFS-full-1	125 kN	(1) Yielding in gross sectional area; (2) debonding of CFRP; (3) yielding at breakage in net area.
CFS-full-2	127 kN	
CFS-loss-1	126 kN	
CFS-loss-2	127 kN	

## Data Availability

The data presented in this study are available on request from the corresponding author.

## Supplementary Materials

The following are available online at https://www.mdpi.com/article/10.3390/ma14092184/s1, Figure S1: Experimental video of CFRP strengthening specimen.
