# Supplementary material for: Pilot Demonstration of a Strengthening Method for Steel-Bolted Connections Using Pre-Formable Carbon Fiber Cloth with VaRTM
Source: Materials (Basel). 2021 Apr 24;14(9):2184. doi: 10.3390/ma14092184 (PMC8123189; doi:10.3390/ma14092184)
Supplement: Supplementary file 1 [file materials-14-02184-s001.zip › Supplementary materials-1164316.pdf]

materials

MDPI

Article

Pilot Demonstration of a Strengthening Method for Steel-Bolted Connections Using Pre-Formable Carbon Fiber Cloth with VaRTM

Takahiro Matsui [1], Kohei Suzuki [2], Sota Sato [2], Yuki Kubokawa [3], Daiki Nakamoto [3], Shijir Davaakhishig [3] and Yukihiro Matsumoto [3,*]

1 ACM Technology Dept., Toray Industries, Inc., 2-1-1 Nihonbashimuromachi, Chuo-ku, Tokyo 103-8666, Japan; takahiro.matsui.f3@mail.toray

2 Technical Development Headquarters, Constec Engi, Co., Ltd., 6-1-1 Heiwajima, Ota-ku, Tokyo 143-0006, Japan; suzuki-kohei@cons-hd.co.jp (K.S.); sato-sota@cons-hd.co.jp (S.S.)

3 Department of Architecture and Civil Engineering, Toyohashi University of Technology, 1-1 Hibarigaoka, Tempaku-cho, Toyohashi, Aichi 441-8580, Japan; kubokawa.yuki.vo@tut.jp (Y.K.); nakamoto.daiki.md@tut.jp (D.N.); shijir.davaakhishig.hj@tut.jp (S.D.)

* Correspondence: y-matsum@ace.tut.ac.jp; Tel.: +81-532-44-6845

Citation: Matsui, T.; Suzuki, K.; Sato, S.; Kubokawa, Y.; Nakamoto, D.; Davaakhishig, S.; Matsumoto, Y. Pilot Demonstration of a Strengthening Method for Steel-Bolted Connections Using Pre-Formable Carbon Fiber Cloth with VaRTM. *Materials* **2021**, *14*, x. https://doi.org/10.3390/xxxxx

Academic Editor: Jose Sena-Cruz

Received: 15 March 2021
Accepted: 21 April 2021
Published: 24 April 2021

Publisher's Note: MDPI stays neutral with regard to jurisdictional claims in published maps and institutional affiliations.

Copyright: © 2021 by the authors. Licensee MDPI, Basel, Switzerland. This article is an open access article distributed under the terms and conditions of the Creative Commons Attribution (CC BY) license (http://creativecommons.org/licenses/by/4.0/).

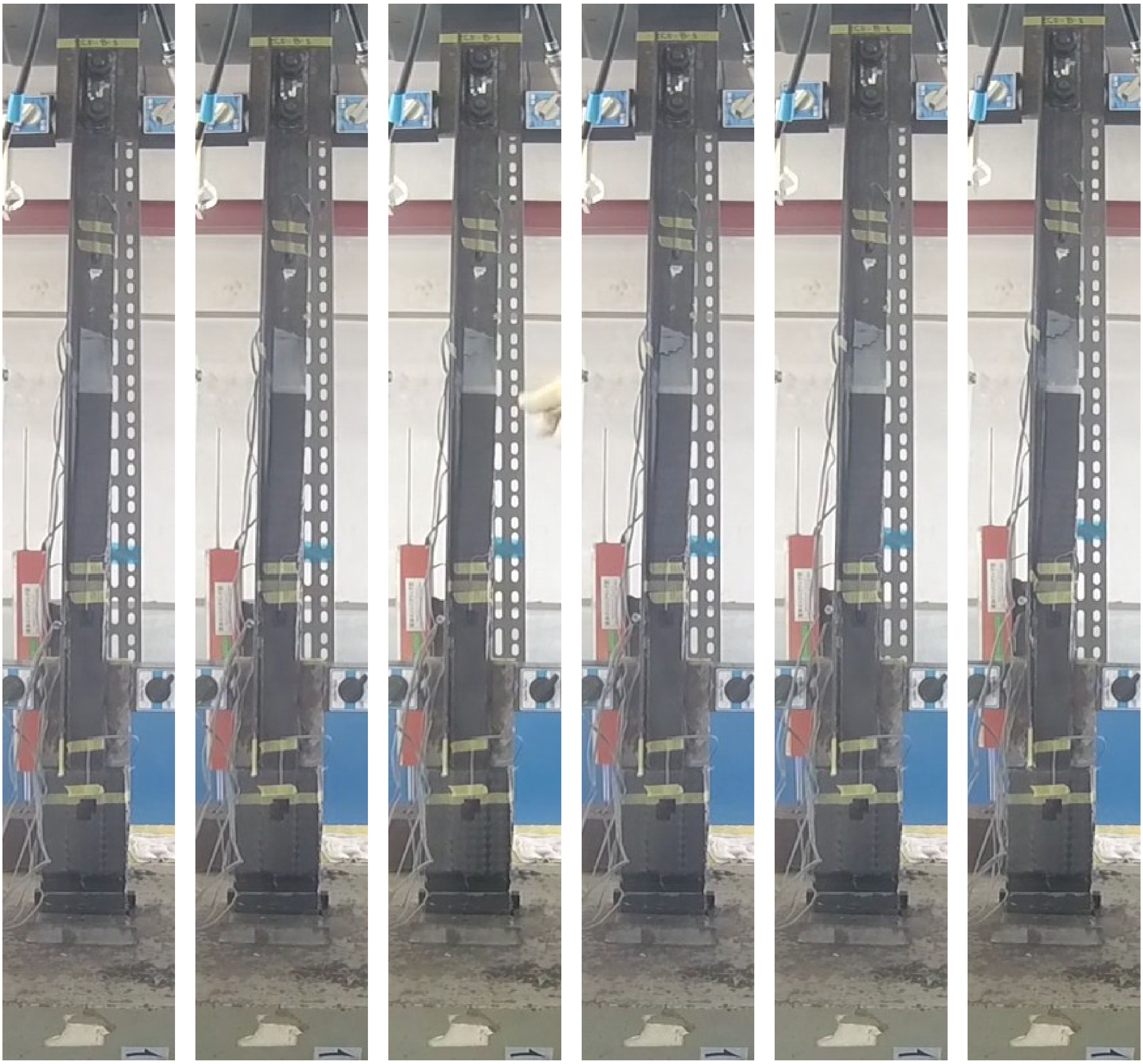

Figure S1. Experimental video of CFRP strengthening specimen.
